# The slope of cerebral oxyhemoglobin oscillation is associated with vascular reserve capacity in large artery steno-occlusion

**DOI:** 10.1038/s41598-021-88198-4

**Published:** 2021-04-21

**Authors:** Tae Jung Kim, Jae-Myoung Kim, Soo-Hyun Park, Jong-Kwan Choi, Hyeon-Min Bae, Sang-Bae Ko

**Affiliations:** 1 Department of Neurology, Seoul National University Hospital, 101 Daehak-ro, Jongno-gu, Seoul, 03080 Republic of Korea; 2 Department of Critical Care Medicine, Seoul National University Hospital, Seoul, Republic of Korea; 3 Department of Neurology, Seoul National University College of Medicine, 103 Daehak-ro, Jongno-gu, Seoul, 03080 Republic of Korea; 4 Department of Research and Development, Optics Brain Electronics Laboratory, OBELAB Inc, Seoul, Republic of Korea; 5 Department of Electrical Engineering, Korea Advanced Institute of Science and Technology, Daejeon, Republic of Korea; 6 Department of Neurology, Inha University Hospital, Incheon, Republic of Korea

**Keywords:** Neuroscience, Medical research, Neurology, Optics and photonics

## Abstract

Inadequate cerebral perfusion is a risk factor for cerebral ischemia in patients with large artery steno-occlusion. We investigated whether prefrontal oxyhemoglobin oscillation (ΔHbO_2_, 0.6–2 Hz) was associated with decreased vascular reserve in patients with steno-occlusion in the large anterior circulation arteries. Thirty-six patients with steno-occlusion in the anterior circulation arteries (anterior cerebral artery, middle cerebral artery, and internal carotid artery) were included and compared to thirty-six control subjects. Patients were categorized into two groups (deteriorated vascular reserve vs. preserved vascular reserve) based on the results of Diamox single- photon emission computed tomography imaging. HbO_2_ data were collected using functional near-infrared spectroscopy. The slope of ΔHbO_2_ and the ipsilateral/contralateral slope ratio of ΔHbO_2_ were analyzed. Among the included patients (n = 36), 25 (69.4%) had deteriorated vascular reserve. Patients with deteriorated vascular reserve had a significantly higher average slope of ΔHbO_2_ on the ipsilateral side (5.01 ± 2.14) and a higher ipsilateral/contralateral ratio (1.44 ± 0.62) compared to those with preserved vascular reserve (3.17 ± 1.36, *P* = 0.014; 0.93 ± 0.33, *P* = 0.016, respectively) or the controls (3.82 ± 1.69, *P* = 0.019; 0.94 ± 0.29, *P* = 0.001). The ipsilateral/contralateral ΔHbO_2_ ratio could be used as a surrogate for vascular reserve in patients with severe steno-occlusion in the anterior circulation arteries.

## Introduction

Poor collateral circulation is one of the risk factors for cerebral infarction in patients with large artery steno-occlusion^1–5^. Recent advancement in multimodal neuroimaging allows better identification of the impairment in cerebral perfusion. However, neuroimaging only provides a snap-shot of information on cerebral hemodynamics and has safety concerns regarding contrast agents or radiation exposure^5–7^. Cerebral near-infrared spectroscopy (NIRS) is a non-invasive monitoring tool to evaluate cerebral oxyhemoglobin (HbO2) and deoxyhemoglobin (HbR). Functional NIRS (fNIRS) has more advantages in spatial resolution compared to electroencephalography or cerebral oximetry devices, which can provide information on regional cerebral oxygenation and hemodynamics at the level of the cortical microcirculation using non-invasive monitoring methods^6–16^.

The HbO2 value of NIRS measurement could provide cerebral perfusion status if cerebral metabolic status remains constant^13, 17^. In addition, the pulse wave of cerebral HbO2 signal, measured by fNIRS, oscillates in phase with arterial pulse waves^17–19^. Previous studies showed that changes of amplitude or phase shift of the arterial pulse wave of the cerebral microvascular oscillation measured by  NIRS  were associated with impaired cerebral perfusion or hypoxia^17–19^. Therefore, these oscillations of HbO2 (ΔHbO2, 0.6-2 Hz) could be regarded as a surrogate for vascular compliance and cerebral autoregulation in cerebral microvessels, especially in arterioles^17–25^. Pulse wave signal analysis may provide information on vascular tones; a steeper slope suggests a state of vasodilation, arterial stiffness, and vascular complicance^17,26–28^. As regional cerebral perfusion pressure decreases due to large vessel steno-occlusion, distal arterioles dilated to maintain cerebral blood flow and oxygenation^29,30^. The slope analysis of ΔHbO2 may differentiate patients with or without vascular reserve capacity among those with large vessel steno-occlusion^17–19,26–28^. Therefore, we hypothesized that the slope of ΔHbO2 signals from the fNIRS could be used to identify patients with cerebral hemodynamic failure.

## Materials and methods

### Study population

Between June 2016 and August 2019, we consecutively recruited 70 patients who were admitted or visited outpatient clinics with the diagnosis of stenosis ≥ 50% or occlusion in the anterior circulation large arteries. The detailed inclusion criteria were as follows: 1) moderate to severe stenosis (≥ 50%) in the anterior cerebral artery (ACA), middle cerebral artery (MCA), and/or intracranial internal carotid artery (ICA)^31^, or 2) moderate to severe stenosis (≥ 50%) in the extracranial ICA^32^. The degree of arterial stenosis was assessed using magnetic resonance angiography, computed tomography angiography, and/or conventional angiography. Patients were excluded if (1) information on the perfusion status was not available (n = 12), (2) the quality of the fNIRS signal was not sufficient for the analysis due to artifact (n = 7), or (3) arterial stenosis in the bilateral hemisphere (n = 15). Among the total of 36 patients, diagnoses were (1) acute ischemic stroke/transient ischemic attack (TIA) in the territory of stenosis (n = 12), (2) a previous history of ischemic stroke/TIA in the territory of stenosis (n = 18), and (3) asymptomatic steno-occlusion (n = 15). For the control group, 36 healthy subjects without evidence of cerebrovascular disease were enrolled. Informed consent was obtained from all participants. All subjects underwent fNIRS monitoring in a supine position for at least 5 min using the previous reported using a wireless continuous-wave near-infrared spectroscopy (CW-NIRS) system (NIRSIT, OBELAB Inc., Seoul, Republic of Korea)^9^. This study was approved by the Institutional Review Board (IRB) of Seoul National University Hospital (IRB Number; H-1606–024-768). In addition, this study was performed in accordance with relevant guidelines and regulations.

### Assessment of cerebrovascular reserve capacity

Cerebral perfusion was assessed using brain single-photon emission computed tomography (SPECT). Deteriorated cerebrovascular reserve was defined using basal/acetazolamide stress brain SPECT, as described previously^33^. Among the included patients, 58.3% (21/36) patients underwent transcranial Doppler (TCD) ultrasonography (Spencer PMD150, USA) using standard protocols, and the pulsatility index (PI) was assessed to evaluate vascular compliance in large cerebral arteries^34^. We retrospectively collected the monitoring data of TCD using electronic medical records. The PI was calculated using the following formula: PI = (systolic flow velocity-diastolic flow velocity)/mean flow velocity^34,35^.

### Baseline patient characteristics and clinical assessments

We assessed baseline characteristics, including age, sex, and conventional vascular risk factors such as hypertension, diabetes mellitus, hyperlipidemia, history of stroke/TIA, coronary artery disease, atrial fibrillation, and smoking status. Neurological assessments were performed on all patients with acute ischemic stroke using the National Institute of Health Stroke Scale (NIHSS) at admission.

### Functional near-infrared spectroscopy measurements

**Figure 1:**
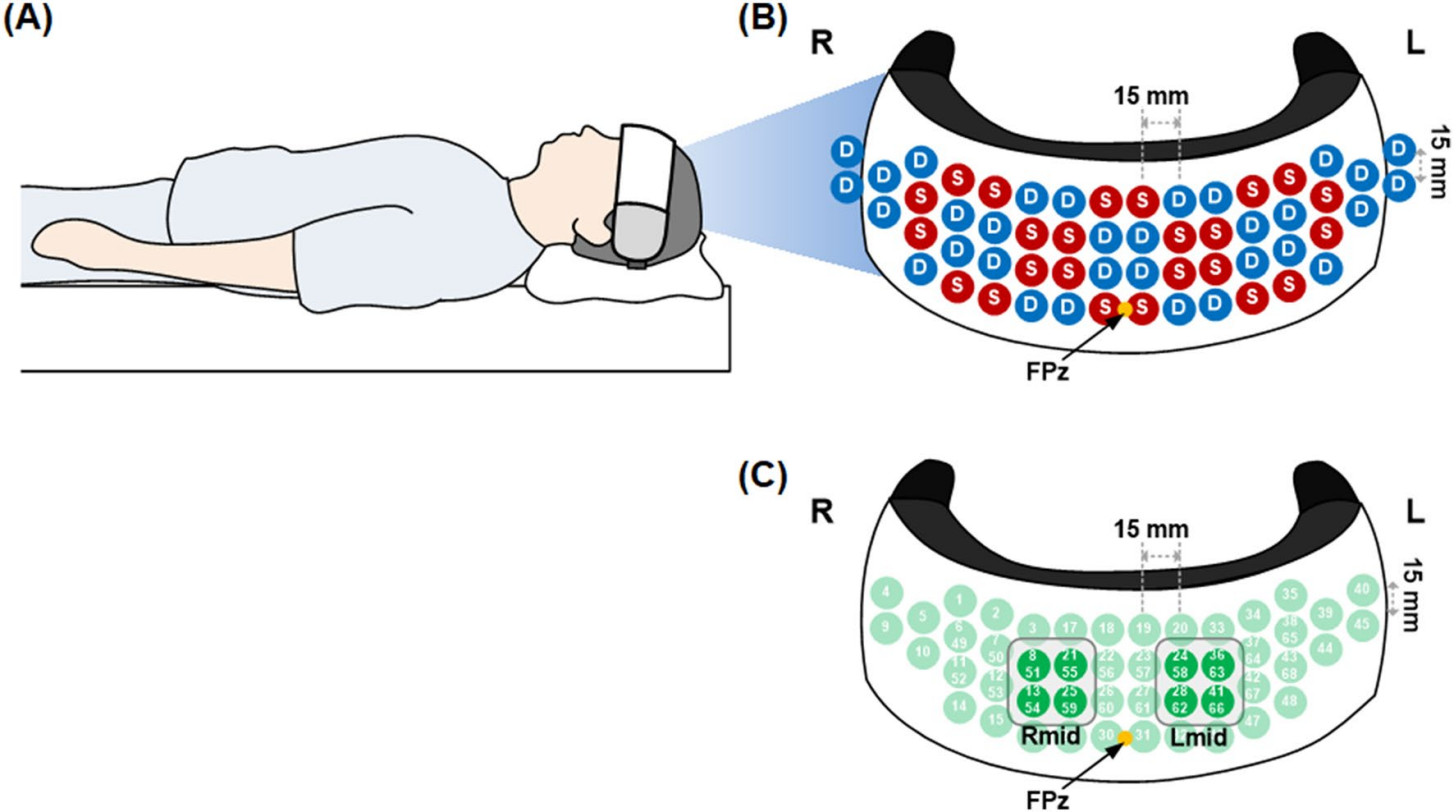
Data acquisition setup using continuous-wave near-infrared spectroscopy and analyzed channels for waveform analysis of oxyhemoglobin. (**A**) Data acquisition setup: The fNIRS signals from the prefrontal lobes were measured for at least 5 min in the supine position. (**B**) Arrangement of sources (red dots) and detectors (blue dots): The center of the lower optical probes is the site of a prefrontal midline electrode (FPz) in the 10–20 EEG system. The optical probes were located at an interval of 1.5 cm. (**C**) Selected channels for analysis: Among total channels, 16 channels were selected from the middle part of the left and right regions in the forehead that mostly had robust signals from the detectors considering the variations in the size and shape of the forehead. R, right hemisphere; L, left hemisphere; R_mid,_ right middle; and L_mid,_ left middle.

The fNIRS signals from the prefrontal lobes were measured for at least 5 min in the supine position in all study subjects using previously described NIRSIT device^9^. Figure 1 (A) depicts the fNIRS data acquisition setup. The CW-NIRS system measures the variation in hemodynamics by utilizing near-infrared laser sources at wavelengths of 780 nm and 850 nm. Optical density changes for each wavelength due to oxygenation variations from the cerebral cortex were sampled at a frequency of Fs = 8.138 Hz. HbO_2_ and HbR were calculated based on the modified Beer-Lambert’s law (MBLL)^25^. The center of the lowermost optical probes accurately corresponded to a prefrontal midline electrode (FPz) in the 10–20 EEG system to maintain an identical sensor position on the scalps of all participants (Fig. 1B). The optical probes were arranged at a distance of 1.5 cm, as shown in Fig. 1B, and the signals were acquired from a total of 68 predefined channels with a source-detector distance of 3 cm (Fig. 1), which allows near-infrared light to penetrate deep enough to measure HbO_2_ and HbR in the cerebral cortex; approximately 15—20 mm deep from the scalp^9,15,36–38^.

### Data processing and waveform analysis of oxyhemoglobin

Data processing was performed using MATLAB (R2018a; Mathworks, Natick, USA). Because of the large variations in the coverage of the forehead due to the small forehead sizes among the elderly participants compared to the younger adults (150.36 ± 17.97 vs. 145.8 ± 16.74 mm, head width of the Korean adults in 20 s vs. 60 s, respectively)^39^, 16 channels were selected from the forehead that mostly had robust signals from the detectors (i.e., R_mid_ channels: 8, 13, 21, 25, 50, 51, 54 and 55; L_mid_ channels: 24, 28, 36, 41, 52, 53, 62, and 66 as shown in Fig. 1C). Then, signal integrity (SI) was evaluated for each channel by calculating the logarithmic ratio between the mean and the standard deviation (SD) from the motionless baseline raw signal for 15 s (i.e., SI = 20·log_10_[mean/SD] dB). Finally, channels with good signal quality (automatic motion artifact identification [SI greater than 30 dB, common procedure for multi-channel NIRS measurement^40–42^]) were selected for further data processing. Channels with abrupt spikes or baseline shifts caused by motion artifacts such as facial movements were manually excluded^43^. The proportion of the signals discarded after the signal quality check was average 9.1% (mean, 1.45; standard deviation [SD], 2.14) from measured 16 channels. The selected signals were low-pass filtered using a discrete cosine transform-based filter with a cutoff frequency of 3 Hz only to obtain pulsatile oscillations by eliminating the high-frequency noise. Finally, MBLL was applied for each channel to extract the HbO_2_ concentration changes.

**Figure 2:**
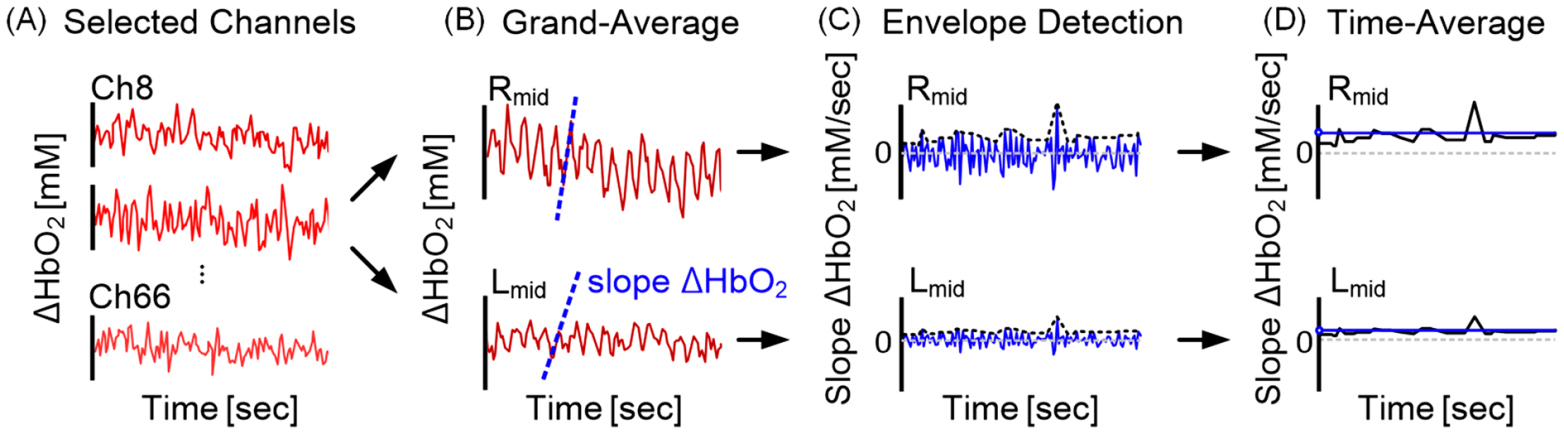
Typical example of data processing procedure. (**A**) Original ΔHbO2 signal of selected 16 channels of two included subjects from each group. (**B**) Grand averaged waveform of the HbO2 signals from the selected 16 channels into the left and right hemispheres to increase the integrity of the pulsatile oscillation using the synchronized response of averaged ΔHbO_2_ (**C**) Envelope detection of maximum slope values of ΔHbO_2_ in each hemisphere by time-differentiating between the two adjacent values of ΔHbO_2_ reflecting the steepness of the pulsation at each sampling points (**D**) Average slope value calculated from the envelope of the slope ΔHbO_2_.

As described in Fig. 2A,B, the HbO_2_ signals were averaged for each R_mid_ and L_mid_ regions to increase the integrity of the pulsatile oscillation by averaging the synchronized oscillatory response of ΔHbO_2_. The slope value of ΔHbO_2_ was acquired by time-differentiating between the two adjacent values of ΔHbO_2_ reflecting the steepness of the pulsation at each sampling point in each hemisphere (Fig. 2C). The upper envelope was detected from the oscillation of the slope value of ΔHbO_2_ by extracting the local maximum values over a sliding window which represents the sharpness of the ΔHbO_2_ wave. Finally, the upper envelope was averaged into a single representative slope value for the left and right hemispheres, respectively (Fig. 2D). The unit of the slope value was expressed in 10^–4^·Fs mM/sec (Fs = 8.138 Hz as the sampling frequency).

### Statistical analyses

Patients were categorized into two groups based on the vascular reserve status (decreased vascular reserve vs. intact vascular reserve). Continuous variables including age, initial NIHSS, slope parameters and PI value were compared using Student’s *t*- or Mann–Whitney *U*-tests, and the proportions of categorical variables such as sex, hypertension, diabetes mellitus, hyperlipidemia, coronary artery disease, atrial fibrillation, previous stroke/TIA, smoking, locations of steno-occlusion vessels, and acute ischemic stroke during monitoring were compared using Pearson’s χ^2^ or Fisher’s exact tests, as appropriate. For slope analysis of ΔHbO_2_ oscillation, the slope values and the ipsilateral/contralateral slope ratios were compared among the three groups (decreased vascular reserve vs. intact vascular reserve vs. controls) using the Kruskal–Wallis test or one-way analyses of variance (ANOVA), as appropriate. Data are presented as means ± SDs or as medians with interquartile ranges (IQR), according to the distribution of the data. All variables with *P* < 0.05 were considered statistically significant. All statistical analyses were performed using SPSS (version 25.0; IBM Statistics, Armonk, NY, USA) and GraphPad Prism (Version 8, GraphPad Software, San Diego, CA, USA).

## Results

Among the included patients in this study (n = 36, mean age, 62.3 years; male, 61.1%), 25 (69.4%) had an impairment in vascular reserve based on criteria using Diamox SPECT.

The locations of steno-occlusive vessels were MCA (n = 7, 19.4%), intracranial ICA (n = 4, 11.1%), and extracranial ICA (n = 25, 69.4%), and the proportion of locations was similar between two groups (Table 1). Regarding the vascular risk factors, there was no significant difference between groups with preserved and deteriorated vascular reserve. The control groups (n = 36; male, 50.0%) tended to be younger (mean age, 55.5 years) compared with the study groups (*P* = 0.086). Among the patients, 33.3% (12 out of 36) had an acute ischemic stroke (median 3 days after symptom onset, IQR 0.8–5.5). However, the percentage of patients with acute ischemic stroke did not differ between those with preserved and deteriorated vascular reserve (40.0% vs. 18.2%, respectively, *P* = 0.268, Table 1). In the subgroups with acute ischemic stroke, patients with deteriorated vascular reserve had a slightly higher initial NIHSS (median  2.5 , IQR 0.75–12.75) compared with those with preserved vascular reserve (median 0, IQR [0.0–0.0]) (*P* = 0.080) (Table 1).

**Table 1:** Clinical characteristics of study population.

	Total (n = 36)	Deteriorated vascular reserve (n = 25, 69.4%)	Preserved vascular reserve (n = 11, 30.6%)	P value
Age (mean ± SD), years	62.3 ± 14.0	61.0 ± 15.0	65.3 ± 11.6	0.407
Male, n (%)	22 (61.1)	14 (56.0)	8 (72.7)	0.467
Hypertension, n (%)	23 (63.9)	15 (60.0)	8 (72.7)	0.708
Diabetic mellitus, n (%)	13 (36.1)	10 (40.0)	3 (27.3)	0.708
Hyperlipidemia, n (%)	20 (55.6)	12 (48.0)	8 (72.7)	0.277
CAD, n (%)	8 (22.2)	4 (16.0)	4 (36.4)	0.214
Atrial fibrillation, n (%)	3 (8.3)	3 (12.0)	0 (0.0)	0.538
Previous stroke/TIA, n (%)	18 (50.0)	11 (44.0)	7 (63.6)	0.278
Smoking, n (%)	16 (44.4)	11 (44.4)	5 (45.5)	1.000
Locations of steno-occlusion artery, n (%)				0.394
MCA	7 (19.4)	4 (16.0)	3 (27.3)	
Intracranial ICA	4 (11.1)	4 (16.0)	0 (0.0)	
Extracranial ICA	25 (69.4)	17 (68.0)	8 (72.7)	
Acute ischemic stroke during monitoring, n (%)	12 (33.3)	10 (40.0)	2 (18.2)	0.268
Initial NIHSS, median (IQR) (n = 12)	2 (0–10.75) (n = 12)	2.5 (0.75–12.75) (n = 10)	0 (0–0) (n = 2)	0.080

*SD* standard deviation; *CAD* coronary artery disease; *TIA* transient ischemic attack; *MCA* middle cerebral artery; *ICA* internal carotid artery; *NIHSS* National Institute of Health Stroke Scale; *IQR* interquartile ranges.

**Figure 3:**
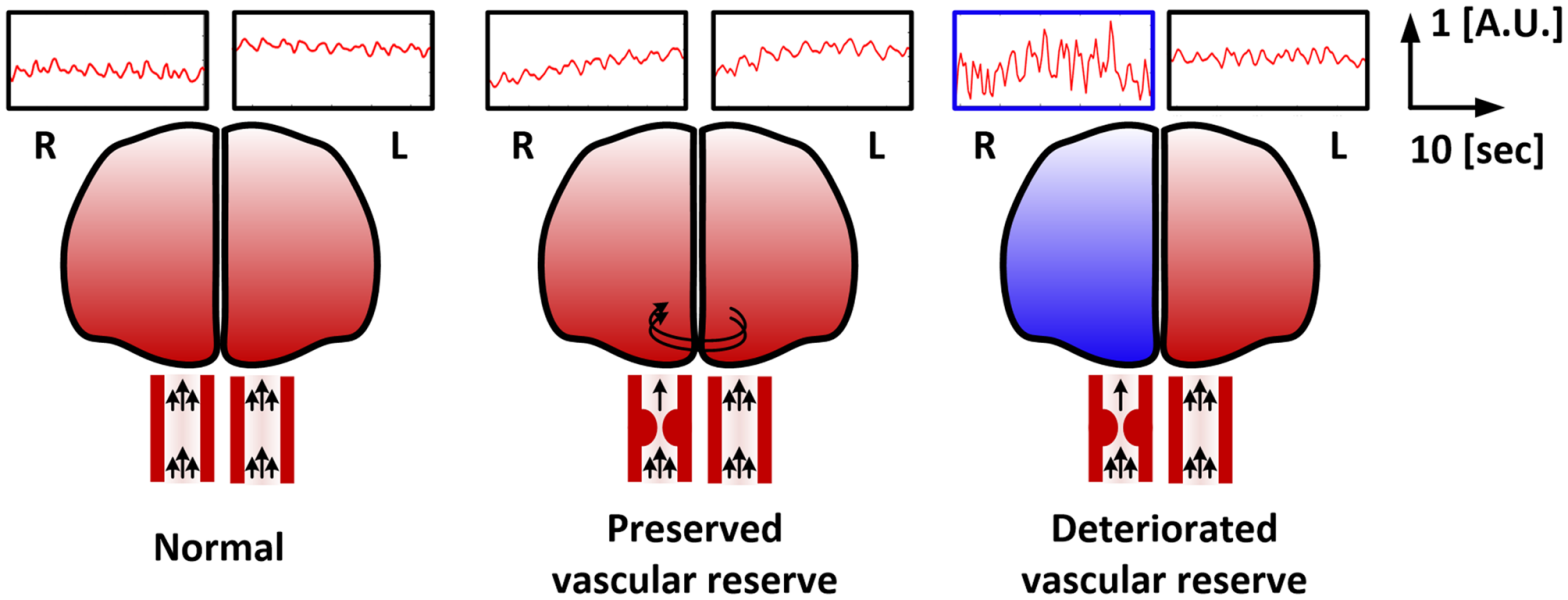
Illustrated waveform of oxyhemoglobin according to the vascular reserve. ΔHbO_2_ wave was steeper on ipsilateral to steno-occlusive vessel in patients with deteriorated vascular reserve compared to contralateral side. This asymmetry was not present in patients with intact vascular reserve or controls. This was drawn using three included subjects from each group.

**Figure 4:**
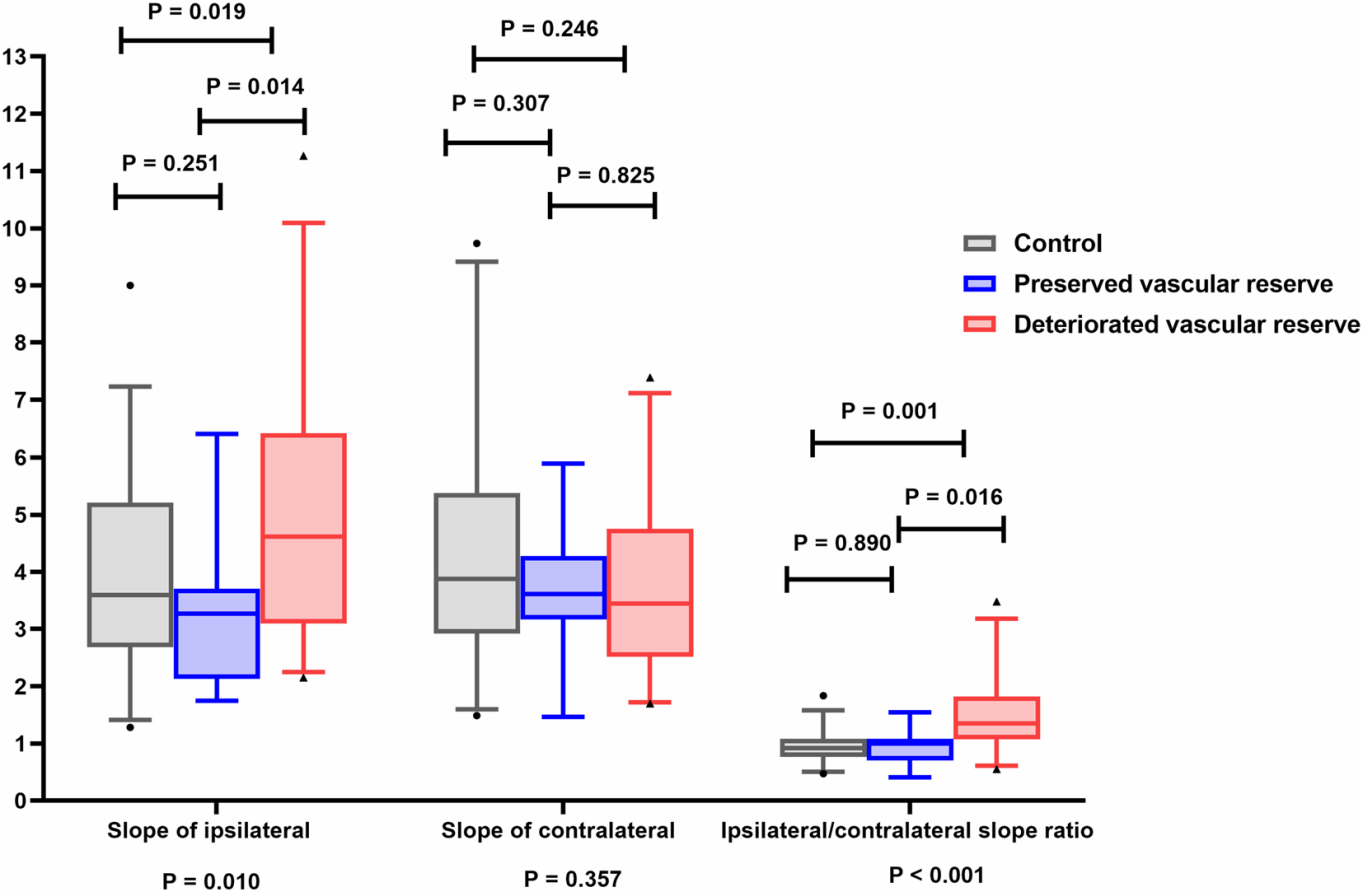
Comparison of the oxyhemoglobin slope wave according to the vascular reserve. The ipsilateral average slope of ΔHbO_2_ and the ipsilateral/contralateral slope ratio was significantly higher in patients with deteriorated vascular reserve compared to those with preserved vascular reserve or controls. However, the slope value did not differ on the contralateral side among the patients and controls. (Box-and-Whisker Plot with 5–95 percentile).

The slope of ΔHbO2 wave analyses showed that the average slope of ΔHbO_2_ on the ipsilateral side to the stenotic vessel was significantly higher in patients with deteriorated vascular reserve (5.01 ± 2.14) compared with those with preserved vascular reserve (3.17 ± 1.36, *P* = 0.014) or controls (3.82 ± 1.69, *P* = 0.019) (Table 2, Figs. 3, 4). However, the slope of ΔHbO_2_ on the contralateral side to the stenotic vessel did not differ among the patients with preserved vascular reserve or with deteriorated vascular reserve or controls (mean ± SD, 3.62 ± 1.24 vs. 3.73 ± 1.52 vs. 4.31 ± 2.10, respectively, *P* = 0.357, ANOVA test, Table 2). Moreover, the SD values of slope of ΔHbO2 in ipsilateral and contralateral sides were not different among the patients with or without preserved vascular reserve and control groups (ipsilateral side [mean ± SD]; 1.01 ± 0.41 vs. 1.36 ± 0.66 vs. 1.41 ± 0.61, respectively, *P* = 0.198, contralateral side [mean ± SD]; 1.27 ± 0.62 vs. 1.04 ± 0.60 vs. 1.27 ± 0.73, respectively, *P* = 0.383, ANOVA test and Table 2). However, the ipsilateral to contralateral slope ratio of ΔHbO_2_ was significantly higher in patients with decreased vascular reserve (1.44 ± 0.62) compared with those with intact vascular reserve (0.93 ± 0.33, *P* = 0.016) or control groups (0.94 ± 0.29, P = 0.001) (Table 2 and Fig. 4). To identify whether the slope of ΔHbO_2_ is a simple reflection of pulsatile perfusion in large vessels, the PI values from 21 patients who underwent TCD were analyzed. However, the PI values in the ipsilateral (0.84 ± 0.23 vs. 0.77 ± 0.21, *P* = 0.537), contralateral (0.83 ± 0.25 vs. 0.83 ± 0.23, *P* = 1.000), and ipsilateral to contralateral PI ratio (1.02 ± 0.17 vs. 0.95 ± 0.17, *P* = 0.376) were not significantly different among the patients with preserved vascular reserve and those with deteriorated vascular reserve (Table 2).

**Table 2:** The value of slope of oxyhemoglobin wave according to cerebral perfusion.

	Control (n = 36)	Deteriorated vascular reserve (n = 25, 69.4%)	Preserved vascular reserve (n = 11, 30.6%)	P value
Slope on the ipsilateral lesion side (mean ± SD)	3.82 ± 1.69	5.01 ± 2.14	3.17 ± 1.36	0.010
Slope on the contralateral side (mean ± SD)	4.31 ± 2.10	3.73 ± 1.52	3.62 ± 1.24	0.357
SD of slope on the ipsilateral lesion side (mean ± SD)	1.14 ± 0.60	1.36 ± 0.66	1.01 ± 0.41	0.198
SD of slope on the contralateral side (mean ± SD)	1.27 ± 0.73	1.04 ± 0.60	1.27 ± 0.62	0.383
Ipsilateral /contralateral slope ratio (mean ± SD)	0.94 ± 0.29	1.44 ± 0.62	0.93 ± 0.33	< 0.001
PI on the ipsilateral lesion side (mean ± SD)	–	0.77 ± 0.21 (n = 14)	0.84 ± 0.23 (n = 7)	0.537
PI on the contralateral lesion side (mean ± SD)	–	0.83 ± 0.23 (n = 14)	0.83 ± 0.25 (n = 7)	1.000
Ipsilateral /contralateral PI ratio (mean ± SD)	-	0.95 ± 0.17 (n = 14)	1.02 ± 0.17 (n = 7)	0.376

*SD* standard deviation; *PI* pulsatility index.

Unit = Fs·10–4 mM/sec; Fs = 8.138 Hz.

The slope of ΔHbO2 wave analyses showed that the average slope of ΔHbO_2_ on the ipsilateral to the stenotic vessel was significantly higher in patients with decreased vascular reserve (5.01 ± 2.14) compared with those with preserved vascular reserve (3.17 ± 1.36, *P* = 0.014).

In patients with acute ischemic stroke (n = 12), the ipsilateral to the contralateral ratio of ΔHbO_2_ did not statistically differ between patients with a preserved vascular reserve and those with deteriorated vascular reserve, probably due to a small sample size (Table 3).

**Table 3:** The slope of oxyhemoglobin wave according to vascular reserve among the patients with acute ischemic stroke (n = 12).

	Deteriorated vascular reserve (n = 10, 83.3%)	Preserved vascular reserve (n = 2, 16.7%)	P value
Slope on the ipsilateral lesion side (mean ± SD)	5.74 ± 2.68	3.07 ± 1.75	0.396
Slope on the contralateral lesion side (mean ± SD)	4.09 ± 1.70	2.65 ± 1.28	0.215
Ipsilateral slope/contralateral slope ratio (mean ± SD)	1.42 ± 0.44	1.13 ± 0.11	0.288

*SD* standard deviation.

Unit = Fs·10–4 mM/sec; Fs = 8.138 Hz.

Among the included patients, one patient had severe stenosis in the right proximal extracranial ICA with an impairment in vascular reserve and underwent stent placement in the carotid artery. ΔHbO_2_ was measured before and after carotid stenting. The average slope of ΔHbO_2_ on the ipsilateral side was higher compared to the contralateral side (right/left 6.90 [SD 1.65]/4.68 [SD 1.17] and ipsilateral to contralateral slope ratio 1.48, systolic blood pressure [SBP]/diastolic blood pressure [DBP]/mean blood pressure [MBP] 130/79/96 mmHg) before carotid stenting. However, the ipsilateral to contralateral ΔHbO_2_ slope ratio decreased after carotid stenting in that patient (right/left 7.76 [SD 2.08]/7.02 [SD 1.38] and ipsilateral to contralateral slope ratio 1.11, SBP/DBP/MBP 109/61/77 mmHg), suggesting an improvement in a vascular reserve capacity. Follow-up Diamox SPECT showed the vascular reserve was improved on the side of stent placement.

## Discussion

In the present study, a higher ipsilateral to contralateral slope ratio of ΔHbO_2_ wave, measured by fNIRS in prefrontal areas, was associated with a decreased vascular reserve in the steno-occlusion in the anterior circulation arteries. Moreover, the ratio decreased after revascularization procedures in one patient who underwent carotid stenting.

NIRS can monitor cerebral HbO_2_ and HbR, and thus indirectly provides information on oscillation of cerebral cortical oxygenation and hemodynamics^17–22, 44–48^. In the analyses, we showed that the ΔHbO_2_ slope on the ipsilateral side to the significant steno-occlusive artery was higher in patients with deteriorated vascular reserve compared to those with preserved vascular reserve or controls, suggesting a possible role of ΔHbO_2_ as a surrogate marker of vascular reserve capacity and vascular compliance. Considering the individual variation in the absolute slope values, ipsilateral to the contralateral ratio of ΔHbO_2_ was more robust to differentiate the patients with a deteriorated vascular reserve from those with preserved vascular reserve.

We think that the steep slope values in patients with a deteriorated vascular reserve are mediated by poor cerebral perfusion and vasodilation. Pulse waves are generated by pulsatile flow, and the slopes could be affected by vascular tone, arterial stiffness and vascular compliance^17, 26–28^. If a flow is compromised, compensatory vasodilation occurs to maintain perfusion, which might lead to a steeper slope of pulse wave^17–19, 49–51^. The absolute slope value may also be affected by systemic blood pressure, thus are prone to individual variation. In order to adjust this, we compared ipsilateral to contralateral slope ratio among the patients with deteriorate vascular reserve, preserved reserve, and controls. As expected, a higher ipsilateral to contralateral slope ratio was observed in patients with deteriorated vascular reserve. Moreover, we also monitored the ipsilateral to the contralateral ratio of ΔHbO_2_ in a patient who had severe carotid stenosis. The slope ratio was measured before and after the procedure and showed that a high slope ratio improved after the successful placement of the carotid stent^10, 11, 17–22, 51–55^. We cannot draw a conclusion based on this single observation. However, further studies are needed to confirm this relationship.

Standard perfusion imaging tools such as perfusion MRI, CT perfusion, or brain SPECT provide the information on vascular characteristics, but do not measure changes in the concentration of oxyhemoglobin and deoxyhemoglobin^5–7^. In addition to the risk of radiation or contrast agents, one of the limitations of these standard imaging methods is that they only provide a snapshot of perfusion. However, bedside fNIRS is a non-invasive tool, thus it allows continuous monitoring for microcirculatory cerebral hemodynamics^46, 47^. As described above, the slope of ΔHbO_2_ wave was not merely a reflection of macrovascular pulsatile flow, which can be easily assessed by PI from TCD. The patients with deteriorated vascular reserve had a higher slope value on the frontal cortex ipsilateral to the stenosis and had a higher ipsilateral to contralateral slope ratio. However, the PI values were not statistically different between the patients with preserved and deteriorated vascular reserve. Taken together, the fNIRS signals could provide more accurate information on microvascular reserve capacity compared to TCD values^17–19, 52, 53^.

This study has several limitations. First, we could not adjust possible confounders that might have affected CBF and cerebral oxygenation, such as blood pressure and arterial carbon dioxide concentrations^42^, we did not have information on cerebral autoregulation, which could be important in assessing the perfusion status in patients with decreased vascular reserve^10, 45, 48, 53–57^. Third, frontal fNIRS monitoring can only detect changes in the frontal lobe. Fourth, NIRS signals may be affected by scalp blood flow. A distance of 30 mm between source and detector would be accepted for standard cerebral monitoring. Although we do not think this is the main factor, there is a possibility that hemodynamic oscillation was partly affected by extracerebral sources. Fifth, TCD was performed at the discretion of physicians because it was not considered necessary to assess vascular reserve capacity in patients with steno-occlusive vessels. Given the nature of retrospective review of TCD data in this study, a comparison between fNIRS and PI from TCD was performed in 58.3%. Therefore, the analyzed PI data should be interpreted with caution. Sixth, we excluded 9.1% rejected channels for accurate data analysis, this rejected proportion is similar to the previous study^58^. Seventh, we excluded 34 patients (48.6%) with bilateral stenosis or poor NIRS signals. Although we think that we could select more homogenous patients with unilateral stenosis, the results need to be interpreted with caution due to possible selection bias.

In conclusion, the ΔHbO_2_ signal slope ratio can provide information on the microvascular perfusion status in patients with severe steno-occlusion in the anterior circulation arteries. The slope ratio of ΔHbO_2_ could be a novel marker of cerebral hemodynamics, and further large-comprehensive studies are needed to confirm the true relationship between ΔHbO_2_ signal slope and cerebral microvascular autoregulation.

## Data Availability

Data supporting the findings of this study are available from the corresponding author (Sang-Bae Ko) on reasonable request.
