# Author Correction: The slope of cerebral oxyhemoglobin oscillation is associated with vascular reserve capacity in large artery steno-occlusion

**DOI:** 10.1038/s41598-021-95362-3

**Published:** 2021-07-29

**Authors:** Tae Jung Kim, Jae‑Myoung Kim, Soo‑Hyun Park, Jong‑Kwan Choi, Hyeon‑Min Bae, Sang‑Bae Ko

**Affiliations:** 1 Department of Neurology, Seoul National University Hospital, 101 Daehak‑ro, Jongno‑gu, Seoul, 03080 Republic of Korea; 2 Department of Critical Care Medicine, Seoul National University Hospital, Seoul, Republic of Korea; 3 Department of Neurology, Seoul National University College of Medicine, 103 Daehak-ro, Jongno-gu, Seoul, 03080 Republic of Korea; 4 Department of Research and Development, Optics Brain Electronics Laboratory, OBELAB Inc, Seoul, Republic of Korea; 5 Department of Electrical Engineering, Korea Advanced Institute of Science and Technology, Daejeon, Republic of Korea; 6 Department of Neurology, Inha University Hospital, Incheon, Republic of Korea

Correction to: *Scientific Reports* 10.1038/s41598-021-88198-4, published online 21 April 2021

In the original version of this Article Jae‑Myoung Kim was incorrectly affiliated with ‘Department of Neurology, Seoul National University College of Medicine, 103 Daehak-ro, Jongno-gu, Seoul 03080, Republic of Korea’. The correct affiliations are listed below.

Department of Research and Development, Optics Brain Electronics Laboratory, OBELAB Inc, Seoul, Republic of Korea.

Department of Electrical Engineering, Korea Advanced Institute of Science and Technology, Daejeon, Republic of Korea.

The original Article has been corrected.

